# Editorial: Nutrition and sustainable development goal 17: partnerships for the goals

**DOI:** 10.3389/fnut.2024.1480618

**Published:** 2024-10-01

**Authors:** Charoula K. Nikolaou

**Affiliations:** School of Science and Engineering, Natural Resources Institute, University of Greenwich, London, United Kingdom

**Keywords:** sustainable goals, partnerships, nutrition, development, prevention

In the landscape of global development, the Sustainable Development Goals (SDGs) set forth by the United Nations stand as a testament to the collective aspirations of humanity. Among these, Sustainable Development Goal 17 (SDG 17), which emphasizes “Partnerships for the Goals,” holds a particularly critical role. It is the linchpin connecting all other goals, facilitating cooperation and collaboration across borders, sectors, and disciplines. Within this framework, addressing global nutrition challenges emerges as a quintessential area where partnerships can drive substantial progress, impacting multiple SDGs simultaneously.

Nutrition is not an isolated issue; it is intricately linked to various other aspects of development. Malnutrition in multiple forms-undernutrition, micronutrient deficiencies, overweight, and obesity-impacts health, education, economic productivity, and inequality. Thus, tackling nutrition directly influences the achievement of SDG 2 (Zero Hunger), SDG 3 (Good Health and Wellbeing), SDG 4 (Quality Education), and SDG 8 (Decent Work and Economic Growth). Dealing with nutrition challenges requires a multifaceted strategy, making SDG 17′s focus on partnerships vital. Collaborations can combine resources, knowledge, and creative ideas to address nutrition comprehensively, preventing conflicts between different initiatives.

## The role of partnerships in nutrition

1. Public-Private Partnerships (PPPs): One of the most effective ways to address nutrition challenges is through public-private partnerships. Governments can set regulations and provide infrastructure, while private companies bring innovation, efficiency, and investment. For example, implementing the sugar-sweetened beverages (SSB) tax is one example of such a partnership. Brukalo et al., in their article, explored the views of stakeholders toward the impact of the SSB. In their article, Cai et al., analyze the main points included in the Nutrition plan of China. The plan includes some action points for reducing salt and sugar in products and improving dining conditions. Carino et al. looked at policies' role in supporting environmentally sustainable foodservice in healthcare. While policy is an important mechanism, it should be designed considering hospitals' unique challenges.

2. International Collaboration: Nutrition is a global issue requiring global solutions. International organizations such as the World Health Organization (WHO), the Food and Agriculture Organization (FAO), and UNICEF play pivotal roles in setting standards, conducting research, and providing technical assistance. Collaboration among countries can lead to sharing best practices and resources, enhancing the effectiveness of nutrition programs worldwide. Phulkerd et al. looked at the different classification systems from the government, WHO, the Healthier Choice logo and the NOVA system for promoting healthy diets in Thailand.

3. Non-Governmental Organizations (NGOs) and Civil Society: NGOs and civil society organizations are often at the forefront of addressing malnutrition, especially in underserved communities. Their grassroots connections and understanding of local contexts enable them to implement targeted interventions effectively. Partnerships with these organizations can ensure that initiatives are community-driven and sustainable. Thorsen et al. discuss how upcycled foods can be used to reduce hunger and improve the end product's final nutrition profile.

4. Academic and Research Institutions: Research is essential for understanding the complex causes of malnutrition and developing effective interventions. Partnerships with academic and research institutions can provide the evidence base needed to design and implement effective nutrition programs. These institutions can also play a critical role in monitoring and evaluating the impact of interventions, ensuring accountability and continuous improvement. Berry et al. in their opinion piece, also highlight that science is an agent of change.

While partnerships offer immense potential, they are not without challenges. Aligning the diverse interests of various stakeholders can be difficult, and power imbalances can sometimes hinder equitable collaboration. Ensuring that partnerships are inclusive, transparent, and accountable is essential for their success. Moreover, innovative financing mechanisms are needed to support nutrition initiatives. Traditional funding sources often need to be more comprehensive to meet the scale of the challenge. Leveraging new financing models like impact investing and blended finance can mobilize additional resources and drive more sustainable solutions.

To leverage the full potential of partnerships for addressing global nutrition challenges, a few key actions are necessary.

Strengthening Governance: effective governance structures are crucial for managing partnerships. Clear roles, responsibilities, and accountability mechanisms can help ensure effective and equitable partnerships.

Fostering Inclusivity: ensuring that all relevant stakeholders, including marginalized communities, have a voice in partnership initiatives is essential for their success. This inclusivity can enhance the relevance and sustainability of interventions.

Enhancing Capacity Building: building the capacity of local institutions and communities is vital for the long-term success of nutrition initiatives. Partnerships should focus on transferring knowledge and skills to regional actors, empowering them to sustain progress.

Promoting Innovation: encouraging innovation through research and development can lead to new solutions for nutrition challenges. Partnerships should support innovation by providing the necessary resources, creating an enabling environment for experimentation, and scaling up successful interventions.

In conclusion, SDG 17-Partnerships for the Goals-is a cornerstone for achieving improved nutrition and broader sustainable development objectives. By fostering practical, inclusive, and innovative partnerships, the global community can make significant strides in addressing the complex challenges of malnutrition. This collaborative approach enhances the impact of individual efforts. It creates a synergistic effect, propelling us closer to a world where everyone has access to the nutrition they need to lead healthy, productive lives.

